# Dataset supporting the identification of natural dyes in yellow, orange, brown and green fibres from Krakow liturgical vestments

**DOI:** 10.1016/j.dib.2020.105735

**Published:** 2020-05-18

**Authors:** Katarzyna Lech

**Affiliations:** Faculty of Chemistry, Warsaw University of Technology, Noakowskiego 3, 00-664 Warsaw, Poland

**Keywords:** Natural dyes, Colourants, Textiles, High-performance liquid chromatography, Tandem mass spectrometry

## Abstract

This data article provides an extensive and complete description of the colorants and dyes identified in fibre samples taken from the historical textiles that were described in the article "Universal analytical method for characterization of yellow and related natural dyes in liturgical vestments from Krakow” by K. Lech [1]. Natural organic dyes, for centuries used to dye fibres, contain usually from a few to several dyeing compounds. The correct identification of the dye requires at first the identification of their colouring components using sensitive and selective analytical techniques. One of this technique is high-performance liquid chromatography combined with spectrophotometric detection and detection using tandem mass spectrometry with electrospray ionization (HPLC-UV-Vis-ESI MS/MS). The HPLC-UV-Vis-ESI MS/MS protocol was used to identify natural dyes present in 89 yellow, orange, brown and green fibres taken from 15th- to 17th-century silk textiles used in vestments belonging to the collections of seventeen churches in Krakow, Poland.

**Specifications table**

**Table:** 

Subject	Analytical Chemistry
Specific subject area	Identification of yellow, orange and brown dyes natural in historical textiles
Type of data	Raw LC-MS data Table
How data were acquired	High-performance liquid chromatograph (1220 Infinity II LC System, Agilent Technologies) coupled with two spectrophotometric detectors (1220 Compact VWD and 1200 VWD, Agilent Technologies) and tandem mass spectrometric detector (6460 Triple Quad LC/MS with JetStream Technology, Agilent Technologies)
Data format	Raw and analysed
Parameters for data collection	Yellow, orange, brown and green fibres from 15th- to 17th-century silk textiles used in the vestments belonging to the collections of seventeen churches in Krakow
Description of data collection	Fibre extracts were analysed on the presence of natural colourants by HPLC-UV-Vis-ESI MS/MS using positive and negative dynamic multiple reaction monitoring (dMRM) modes. Brown and green fibres were extracted twice with DMSO and with acidic-methanol solution, whereas yellow and orange fibres were extracted only using the second procedure.
Data source location	Faculty of Chemistry, Warsaw University of Technology, Warsaw, Poland
Data accessibility	With the article Repository name: Mendeley Data Data identification number: 10.17632/w24kygn7p8.1 Direct URL to data: https://doi.org/10.17632/w24kygn7p8.1
Related research article	Lech K., Universal analytical method for characterization of yellow and related natural dyes in liturgical vestments from Krakow, Journal of Cultural Heritage (In Press) [1]

## Value of the data

• These data support identification of natural dyes in historical vestments by identification of the colourants using HPLC-UV-Vis-ESI MS/MS
• The dataset informs on natural dyes used in historical textiles from the 15th to the 17th centuries
• These data can be valuable for conservators and art historians
• The dataset increases knowledge on the historical vestments from Krakow churches

## 1 Data Description

Twofold extracts of 89 yellow, orange, brown and green fibres taken from historical textiles have been examined by HPLC-UV-Vis-ESI MS/MS using previously developed method [1] in dynamic multiple reaction monitoring (dMRM) mode. The silk textiles are dated from the 15th to the 17th centuries and they have been used in vestments belonging to the collections of seventeen Krakow churches. The DMSO extracts have been analysed using positive ion mode, whereas methanol-water-formic acid extracts have been examined with both positive and negative ion modes. Identification of the colorants have been based on the compatibility of their retention times and MRM transitions with standards and natural dye markers. Acquired results have led to identification of natural dyes in the examined fibres, even though some of them were re-dyed with synthetic dyes. All samples, their details and attributions, as well as identified colorants and dyes are listed in Table 1. Original dataset is available in the repository [2].

**Table 1:** Colourants and dyes identified in the textile fibres from liturgical vestments (dated fron the 15th to the 17th centuries)

Storage location	Textile number*	Object*	Origin and dating*	Fibre colour	Identified compounds	Original dye	Remarks
Corpus Christi Basilica	No. 1	chasuble, textile from sides	Europe, the 15th cent. ^1^	green	luteolin *C-*hex, carminic acid, **genistin**, **luteolin 7-*****O-*****glc**, genistein *O-*hex, luteolin *O-*hex (1), **apigenin 7-*****O-*****glc**, luteolin *O-*hex (2), *O-*methylluteolin *O-*glcr, **luteolin**, **genistein**, luteolin methyl ether, apigenin, diosmetin, isatin, **indigotin**, indirubin	dyer's broom + indigo/woad	
Church of St Adalbert	No. 2	chasuble, textile from sides	Europe, the 15th cent. ^1^	green	**luteolin 7-*****O-*****hex**, **luteolin*****O-*****glcr(1)**, **luteolin*****O-*****glcr(2)**, *O-*methylluteolin *O-*glcr, **luteolin**, apigenin, isatin, **indigotin**	sawwort + indigo/woad	
Church of St Francis of Assisi (Monastery Church of the Franciscans)	No. 3	chasuble, textile from sides	Europe, the 15th cent. ^1^	green	**luteolin di-*****O-*****hex**, **luteolin 7-*****O-*****glc**, **luteolin*****O-*****hex (1), apigenin 7-*****O-*****glc, luteolin*****O-*****hex (2)**, **luteolin**, apigenin, diosmetin, acacetin, isatin, **indigotin**, indirubin	weld + indigo/woad	
Church of St Francis of Assisi (Monastery Church of the Franciscans)	No. 3	stole, main textile	Europe, the 15th cent. ^1^	green	**luteolin di-*****O-*****hex**, **luteolin 7-*****O-*****glc**, **apigenin 7-*****O-*****glc**, *O-*methylluteolin *O-*glcr, **luteolin**, apigenin, diosmetin, acacetin, isatin, **indigotin**, indirubin	weld + indigo/woad	
Church of St Michael the Archangel and St Stanislaus Bishop and Martyr (Monastery Church of the Pauline Fathers)	No. 4	chasuble, textile from sides	Europe, the 15th cent. ^1^	blueish green	**luteolin di-*****O-*****hex**, **luteolin 7-*****O-*****glc**, apigenin 7-*O-*glc, luteolin *O-*hex (2), *O-*methylluteolin *O-*glcr, **luteolin**, isatin, **indigotin**, indirubin	weld + indigo/woad	
Basilica of Holy Trinity (Monastery Church of the Dominicans)	No. 6	maniple, main textile	Europe, the 15th cent. ^1^	green	l**uteolin di-*****O-*****hex**, **luteolin 7-*****O-*****glc**, apigenin 7-*O-*glc, luteolin *O-*hex (2), **luteolin**, apigenin, diosmetin, isatin, **indigotin**, indirubin	weld + indigo/woad	traces of synthetic dye
Basilica of Holy Trinity (Monastery Church of the Dominicans)	No. 11	stole, main textile	Europe, the 15th cent. ^1^	brown	fustin, ellagic acid, **sulfuretin**, isatin, **indigotin**	young fustic + traces of indigo/woad	
Basilica of Holy Trinity (Monastery Church of the Dominicans)	No. 11	dalmatic, front orphrey	Europe, the 15th cent. ^1^	brown	fustin, ellagic acid, **sulfuretin**	young fustic	
Basilica of Holy Trinity (Monastery Church of the Dominicans)	No. 29	chasuble, textile from sides	Europe, the 15th-16th cent. ^1^	green	luteolin *C-*hex, **genistin**, **luteolin 7-*****O-*****glc**, genistein *O-*hex, luteolin *O-*hex (1), apigenin 7-*O-*glc, luteolin *O-*hex (2), *O-*methylluteolin *O-*glcr, **luteolin, genistein**, luteolin methyl ether, apigenin, isatin, **indigotin**	dyer's broom + indigo/woad	
Church of the Assumption of the Blessed Virgin Mary (Monastery Church of the Camaldolese Monks)	No. 31	chasuble, textile from sides	N/A, the 16th cent. ^1^	green	**luteolin di-*****O-*****hex**, **luteolin 7-*****O-*****glc**, **apigenin 7-*****O-*****glc**, luteolin *O-*hex (2), *O-*methylluteolin *O-*glcr, **luteolin**, apigenin, diosmetin, isatin, **indigotin**, indirubin	weld + indigo/woad	
Basilica of Holy Trinity (Monastery Church of the Dominicans)	No. 52	cope, textile of cope proper	Europe, the 16th cent. ^1^	green	**luteolin di-*****O-*****hex**, **luteolin 7-*****O-*****glc**, luteolin *O-*hex (1), **apigenin 7-*****O-*****glc**, luteolin *O-*hex (2), luteolin, apigenin, diosmetin, isatin, **indigotin**, indirubin	weld + indigo/woad	
Church of St Francis of Assisi (Monastery Church of the Franciscans)	No. 58	stole, main textile	Europe, the 16th cent. ^1^	yellow	digalloylglucose, braz1, **braz2, braz3**, **braz4**, luteolin di-*O-*hex, **braz5**, **luteolin 7-*****O-*****glc**, ellagic acid, apigenin 7-*O-*glc, luteolin *O-*hex (2), luteolin	brazilwood + weld + ellagitannins	
Basilica of Holy Trinity (Monastery Church of the Dominicans)	No. 69	maniple, textile from the endings	Europe, the 16th cent. or c. 1600^1^	green	**luteolin di-*****O-*****hex**, **luteolin 7-*****O-*****glc**, luteolin *O-*hex (1), **apigenin 7-*****O-*****glc**, luteolin *O-*hex (2), **luteolin**, apigenin, diosmetin, isatin, **indigotin**, indirubin	weld + indigo/woad	traces of synthetic dyes
Corpus Christi Basilica	No. 70	chasuble, main textile	Europe, c. 1600 ^1^	green	luteolin *C-*hex, **genistin**, **luteolin 7-*****O-*****glc**, luteolin *O-*hex (2), **luteolin, genistein**, luteolin methyl ether, isatin, **indigotin**, indirubin	dyer's broom + indigo/woad	
Church of St Stephen	No. 72	chasuble, textile from sides	Europe, the 16th cent. ^1^	green	**luteolin di-*****O-*****hex**, **luteolin 7-*****O-*****glc**, apigenin 7-*O-*glc, luteolin *O-*hex (2), **luteolin**, apigenin, isatin, **indigotin**	weld + indigo/woad	
Church of St Stephen	No. 84	chasuble, textile from orphrey	Europe, the 16th cent. ^1^	green	**luteolin di-*****O-*****hex**, **luteolin 7-*****O-*****glc**, apigenin 7-*O-*glc, luteolin *O-*hex (2), **luteolin**, apigenin, isatin, **indigotin**	weld + indigo/woad	
Church of St Barbara	No. 91	burse, main textile	Europe, the 17th cent. 2	green	genistin, **luteolin 7-*****O-*****glc**, **luteolin*****O-*****glcr(1)**, **luteolin*****O-*****glcr(2)**, luteolin *O-*hex (2), *O-*methylluteolin *O-*glcr, **luteolin**, apigenin, isatin, **indigotin**, indirubin	sawwort + indigo/woad + traces of dyer's broom	
Corpus Christi Basilica	No. 96	chalice velum, main textile	Europe, the 17th cent. 2	green	carminic acid, **genistin**, **luteolin 7-*****O-*****glc**, genistein *O-*hex, luteolin *O-*hex (1), **apigenin 7-*****O-*****glc**, luteolin *O-*hex (2), *O-*methylluteolin *O-*glcr, **luteolin, genistein**, luteolin methyl ether, apigenin, biochanin A, isatin, **indigotin**, indirubin	dyer's broom + indigo/woad	
Basilica of Holy Trinity (Monastery Church of the Dominicans)	No. 101	parura, lining	Europe, the 17th cent.	green	luteolin *C-*hex, **genistin**, **luteolin 7-*****O-*****glc**, genistein *O-*hex, luteolin *O-*hex (1), **apigenin 7-*****O-*****glc, luteolin*****O-*****hex (2)**, **luteolin**, genistein, luteolin methyl ether, apigenin, diosmetin, isatin, **indigotin**, indirubin	dyer's broom + indigo/woad	
Church of St Barbara	No. 105	chalice velum, main textile	Europe, the 17th cent.	green	luteolin di-*O-*hex, **luteolin 7-*****O-*****glc**, luteolin *O-*hex (1), **apigenin 7-*****O-*****glc**, luteolin *O-*hex (2), **luteolin**, apigenin, isatin, **indigotin**	weld + indigo/woad	
Corpus Christi Basilica	No. 112	chasuble, main textile	Europe, the 17th cent.	green	**luteolin di-*****O-*****hex**, **luteolin 7-*****O-*****glc**, luteolin *O-*hex (1), apigenin 7-*O-*glc, luteolin *O-*hex (2), *O-*methylluteolin *O-*glcr, **luteolin**, apigenin, diosmetin, isatin, **indigotin**, indirubin	weld + indigo/woad	
Church of St Stephen	No. 116	chasuble, textile from sides	Near East, the 17th cent. 2	green	**luteolin di-*****O-*****hex, genistin**, l**uteolin 7-*****O-*****glc**, **apigenin 7-*****O-*****glc**, luteolin *O-*hex (2), **luteolin**, genistein, apigenin, isatin, **indigotin**	weld + dyer's broom + indigo/woad	
Church of St Mark	No. 118	chasuble, textile from sides	Europe, the 17th cent. 2	green	**luteolin di-*****O-*****hex**, **luteolin 7-*****O-*****glc**, **apigenin 7-*****O-*****glc**, luteolin *O-*hex (2), *O-*methylluteolin *O-*glcr, **luteolin**, apigenin, diosmetin, isatin, **indigotin**	weld + indigo/woad	
Church of St Andrew (Monastery Church of the Poor Clares)	No. 120	stole, main textile	Near East, the 17th cent. 2	green	**luteolin di-*****O-*****hex**, **luteolin 7-*****O-*****glc**, **apigenin 7-*****O-*****glc**, luteolin *O-*hex (2), *O-*methylluteolin *O-*glcr, **luteolin**, apigenin, diosmetin, isatin, **indigotin**	weld + indigo/woad	
Church of St Francis of Assisi (Monastery Church of the Franciscans)	No. 127	stole, main textile	Europe, the 17th cent. 2	green	**luteolin di-*****O-*****hex**, **luteolin 7-*****O-*****glc**, luteolin *O-*hex (1), **apigenin 7-*****O-*****glc**, luteolin *O-*hex (2), *O-*methylluteolin *O-*glcr, **luteolin**, apigenin, diosmetin, isatin, **indigotin**, indirubin	weld + indigo/woad	
Church of St Francis of Assisi (Monastery Church of the Franciscans)	No. 128	maniple, main textile	Europe, the 17th cent. 2	green	**luteolin di-*****O-*****hex**, **luteolin 7-*****O-*****glc**, **apigenin 7-*****O-*****glc**, luteolin *O-*hex (2), *O-*methylluteolin *O-*glcr, **luteolin**, apigenin, diosmetin, isatin, orchil1, orchil2, **indigotin**, indirubin	weld + indigo/woad + traces of orchil	
Basilica of Holy Trinity (Monastery Church of the Dominicans)	No. 130	chasuble, around-neck-opening textile	Europe, the 17th cent. 2	green	**genistin**, **luteolin 7-*****O-*****glc**, **genistein*****O-*****hex**, luteolin *O-*hex (1), apigenin 7-*O-*glc, luteolin *O-*hex (2), **luteolin, genistein**, luteolin methyl ether, apigenin, **biochanin A**, **indigotin**, isatin	dyer's broom + indigo/woad	traces of synthetic dyes
Basilica of Holy Trinity (Monastery Church of the Dominicans)	No. 133	maniple, main textile	Europe, the 17th cent. 2	green	**luteolin di-*****O-*****hex**, **luteolin 7-*****O-*****glc**, luteolin *O-*hex (1), **apigenin 7-*****O-*****glc**, luteolin *O-*hex (2), luteolin, apigenin, diosmetin, isatin, **indigotin**, indirubin	weld + indigo/woad	traces of synthetic dyes
Corpus Christi Basilica	No. 134	chasuble, textile from sides on back	N/A, the 17th cent. 2	green	**genistin**, **luteolin 7-*****O-*****glc**, genistein *O-*hex, luteolin *O-*hex (1), **apigenin 7-*****O-*****glc**, luteolin *O-*hex (2), *O-*methylluteolin *O-*glcr, **luteolin**, **genistein**, luteolin methyl ether, biochanin A, isatin, **indigotin**, indirubin	dyer's broom + indigo/woad	
Basilica of Holy Trinity (Monastery Church of the Dominicans)	No. 140	pall, main textile	Europe, the 17th cent. 2	brown	gallic acid, digalloylglucose, **ellagic acid**, isatin, indigotin	ellagitannins (of unknown origin) + traces of indigo/woad	
Church of St Barbara	No. 143	chasuble, main textile	Europe, the 17th cent. 2	green	**genistin**, **luteolin 7-*****O-*****glc**, genistein *O-*hex, luteolin *O-*hex (1), apigenin 7-*O-*glc, luteolin *O-*hex (2), *O-*methylluteolin *O-*glcr, **luteolin, genistein**, luteolin methyl ether, apigenin, isatin, orchil1, orchil2, **indigotin**	dyer's broom + indigo/woad + traces of orchil	
Basilica of Holy Trinity (Monastery Church of the Dominicans)	No. 145	maniple, main textile	Europe, the 17th cent. 2	green	luteolin *C-*hex, **genistin**, **luteolin 7-*****O-*****glc**, **genistein*****O-*****hex**, luteolin *O-*hex (1), apigenin 7-*O-*glc, luteolin *O-*hex (2), *O-*methylluteolin *O-*glcr, **luteolin, genistein**, luteolin methyl ether, apigenin, isatin, orchil1, orchil2, orchil3, **indigotin**, indirubin	dyer's broom + indigo/woad + traces of orchil	
Corpus Christi Basilica	No. 160	chasuble, main textile	Europe, the 17th cent. 2	green	**luteolin di-*****C-*****hex**, **luteolin 7-*****C-*****glc**, apigenin 7-*C-*glc, luteolin *C-*hex (2), *C-*methylluteolin *C-*glcr, **luteolin**, apigenin, diosmetin, isatin, **indigotin**, indirubin	weld or dyer's broom + indigo/woad	
Church of St Francis of Assisi (Monastery Church of the Franciscans)	No. 161	chalice velum, main textile	Europe, the 17th cent. 2	green	luteolin *C-*hex, **genistin**, **luteolin 7-*****O-*****glc**, genistein *O-*hex, luteolin *O-*hex (1), **apigenin 7-*****O-*****glc**, luteolin *O-*hex (2), *O-*methylluteolin *O-*glcr, **luteolin**, genistein, luteolin methyl ether, apigenin, isatin, **indigotin**, indirubin	dyer's broom + indigo/woad	
Church of St Peter and St Paul	No. 164	cope, textile from hood	Europe, the 17th cent. 2	green	carminic acid, **genistin**, **luteolin 7-*****O-*****glc**, genistein *O-*hex, luteolin *O-*hex (1), apigenin 7-*O-*glc, luteolin *O-*hex (2), **luteolin, genistein**, isatin, **indigotin**, indirubin	dyer's broom + indigo/woad + traces of cochineal (of unknown origin)	traces of synthetic dyes
Church of the Conversion of St Paul (Monastery Church of the Lazarists)	No. 166	cope, main textile	Europe, the 17th cent. 2	green	chlorogenic acid, luteolin *C-*hex, **genistin**, **luteolin 7-*****O-*****glc**, genistein *O-*hex, luteolin *O-*glcr (1), luteolin *O-*glcr (2), **apigenin**7-*O-*glc, *O-*methylluteolin *O-*glcr, **luteolin**, genistein, apigenin, diosmetin, biochanin A, isatin, **indigotin**	dyer's broom + indigo/woad	presence of synthetic dye, original fibre colour – green
Basilica of Holy Trinity (Monastery Church of the Dominicans)	No. 172	stole, main textile	Europe, the 17th cent. 2	green	chlorogenic acid, luteolin *C-*hex, **luteolin 7-*****O-*****glc**, l**uteolin*****O-*****glcr (1)**, **luteolin*****O-*****glcr(2)**, **luteolin**, apigenin, isatin, **indigotin**	sawwort + indigo/woad	
Church of St Anne	No. 175	chasuble, main textile	Europe, the 17th cent. 2	green	**luteolin di-*****O-*****hex, luteolin 7-*****O-*****glc**, luteolin *O-*hex (1), **apigenin 7-*****O-*****glc, luteolin*****O-*****hex (2)**, *O-*methylluteolin *O-*glcr, **luteolin**, apigenin, diosmetin, isatin, **indigotin**, indirubin	weld + indigo/woad	
Corpus Christi Basilica	No. 181	chalice velum, main textile	Europe, the 17th cent. 2	blue	carminic acid, **genistin**, **luteolin 7-*****O-*****glc**, ellagic acid, apigenin 7-*O-*glc, luteolin *O-*hex (2), **luteolin**, **genistein**, luteolin methyl ether, apigenin	dyer's broom	presence of synthetic dye, original fibre colour – yellow
Church of St Joseph (Monastery Church of the Bernardine Nuns)	No. 188	damask, textile fragment	Europe, the 17th cent. 2	green	luteolin *C-*hex, carminic acid, **genistin**, **luteolin 7-*****O-*****glc**, luteolin *O-*glcr (1), luteolin *O-*glcr (2), *O-*methylluteolin *O-*glcr, **luteolin**, genistein, isatin, **indigotin**, indirubin	dyer's broom + indigo/woad	
Church of St Michael the Archangel and St Stanislaus Bishop and Martyr (Monastery Church of the Pauline Fathers)	No. 190	chasuble, textile from sides	Europe, the 17th cent. 2	green	chlorogenic acid, luteolin *C-*hex, genistin, **luteolin 7-*****O-*****glc**, **luteolin*****O-*****glcr (1)**, **luteolin*****O-*****glcr(2)**, *O-*methylluteolin *O-*glcr, **luteolin**, genistein, apigenin, diosmetin, isatin, **indigotin**, indirubin	dyer's broom + sawwort + indigo/woad	
Basilica of Holy Trinity (Monastery Church of the Dominicans)	No. 191	chasuble, textile from sides	Europe, the 17th cent. 2	green	**genistin**, **luteolin 7-*****O-*****glc**, genistein *O-*hex, luteolin *O-*hex (1), **apigenin 7-*****O-*****glc**, luteolin *O-*hex (2), *O-*methylluteolin *O-*glcr, **luteolin, genistein**, luteolin methyl ether, isatin, **indigotin**, indirubin	dyer's broom + indigo/woad	
Basilica of Holy Trinity (Monastery Church of the Dominicans)	No. 195	chalice velum, main textile	Europe, the 17th cent. 2	green	**luteolin di-*****O-*****hex**, **luteolin 7-*****O-*****glc**, luteolin *O-*hex (1), **apigenin 7-*****O-*****glc**, luteolin *O-*hex (2), luteolin, apigenin, diosmetin, isatin, **indigotin**, indirubin	weld + indigo/woad	
Basilica of Holy Trinity (Monastery Church of the Dominicans)	No. 196	stole, textile from the endings	Europe, the 17th cent. 2	red	braz1, braz2, **braz3**, **braz4**, **braz5**, braz6, **bixin**	brazilwood + annatto	presence of synthetic dye, original fibre colour – yellow or orange
Church of St Andrew (Monastery Church of the Poor Clares)	No. 198	chalice velum, main textile	Europe, the 17th cent. 2	green	**genistin**, **luteolin 7-*****O-*****glc**, genistein *O-*hex, luteolin *O-*hex (1), apigenin 7-*O-*glc, luteolin *O-*hex (2), *O-*methylluteolin *O-*glcr, **luteolin, genistein**, luteolin methyl ether, isatin, orchil1, orchil2, orchil3, **indigotin**	dyer's broom + indigo/woad + traces of orchil	
Church of St Barbara	No. 200	chasuble, textile from sides	Europe, the 17th cent. 2	green	**genistin**, **luteolin 7-*****O-*****glc**, genistein *O-*hex, luteolin *O-*hex (1), apigenin 7-*O-*glc, luteolin *O-*hex (2), *O-*methylluteolin *O-*glcr, **luteolin, genistein**, luteolin methyl ether, apigenin, isatin, **indigotin**	dyer's broom + indigo/woad	
Basilica of Holy Trinity (Monastery Church of the Dominicans)	No. 201	chasuble, textile from orphrey and around-neck-opening textile	Europe, the 17th cent. 2	green	luteolin *C-*hex, **genistin**, **luteolin 7-*****O-*****glc**, **genistein*****O-*****hex**, luteolin *O-*hex (1), apigenin 7-*O-*glc, luteolin *O-*hex (2), *O-*methylluteolin *O-*glcr, **luteolin, genistein**, luteolin methyl ether, apigenin, isatin, orchil1, orchil2, **indigotin**	dyer's broom + indigo/woad + traces of orchil	
Church of St Francis of Assisi (Monastery Church of the Franciscans)	No. 206	chasuble, textile from sides	Europe, the 17th cent. 2	green	**luteolin di-*****O-*****hex**, **luteolin 7-*****O-*****glc**, **luteolin*****O-*****hex (1), apigenin 7-*****O-*****glc**, luteolin *O-*hex (2), **luteolin**, apigenin, diosmetin, isatin, orchil1, orchil2, **indigotin**, indirubin	weld + indigo/woad + traces of orchil	
Church of St Francis of Assisi (Monastery Church of the Franciscans)	No. 208	chasuble, textile on back sides and insert in front orphrey	Europe, the 17th cent. 2	yellow	**braz1**, **braz2**, braz3, **braz4**, **braz5**, braz6, **sulfuretin**	brazilwood + young fustic	
Church of St Barbara	No. 208	chasuble, textile from sides	Europe, the 17th cent. 2	orange	braz1, braz2, braz3, **braz4**, carminic acid, genistin, **braz5**, luteolin 7-*O-*glc, genistein, biochanin A	brazilwood + dyer's broom	
Church of St Peter and St Paul	No. 223	stole, main textile	Europe, the 17th cent. 3	red	carminic acid, **genistin**, **luteolin 7-*****O-*****glc**, genistein *O-*hex, luteolin *O-*hex (1), apigenin 7-*O-*glc, luteolin *O-*hex (2), *O-*methylluteolin *O-*glcr, **luteolin, genistein**, luteolin methyl ether, isatin, **indigotin**	dyer's broom + indigo/woad	presence of synthetic dye, original fibre colour – green
Church of St Peter and St Paul	No. 224	chasuble, main textile	Europe, the 17th cent. 3	yellow	luteolin *C-*hex, genistin, **luteolin 7-*****O-*****glc**, genistein *O-*hex, luteolin *O-*hex (1), apigenin 7-*O-*glc, luteolin *O-*hex (2), **luteolin**, **genistein**, isatin, **indigotin**, indirubin	dyer's broom + indigo/woad	
Church of St Anne	No. 227	chasuble, textile from sides	Europe, the 17th cent. 3	green	**genistin**, **luteolin 7-*****O-*****glc**, genistein *O-*hex, luteolin *O-*hex (2), apigenin 7-*O-*glc, **luteolin, genistein**, isatin, **indigotin**, indirubin	dyer's broom + indigo/woad	
Basilica of Holy Trinity (Monastery Church of the Dominicans)	No. 231	chasuble, main textile	Europe, the 17th cent. 3	yellow	**genistin**, **luteolin 7-*****O-*****glc**, **genistein*****O-*****hex**, luteolin *O-*hex (1), **apigenin 7-*****O-*****glc**, luteolin *O-*hex (2), *O-*methylluteolin *O-*glcr, sulfuretin, **luteolin**, **genistein**, **luteolin methyl ether**	dyer's broom + young fustic	
Basilica of Holy Trinity (Monastery Church of the Dominicans)	No. 231	maniple, main textile	Europe, the 17th cent. 3	yellow	**genistin**, **luteolin 7-*****O-*****glc**, genistein *O-*hex, luteolin *O-*hex (1), **apigenin 7-*****O-*****glc**, luteolin *O-*hex (2), *O-*methylluteolin *O-*glcr, sulfuretin, **luteolin**, **genistein**, luteolin methyl ether, **bixin**	dyer's broom + annatto + young fustic	
Basilica of Holy Trinity (Monastery Church of the Dominicans)	No. 232	chalice velum, main textile	Europe, the 17th cent. 3	yellow	**genistin**, **luteolin 7-*****O-*****glc**, genistein *O-*hex, luteolin *O-*hex (1), apigenin 7-*O-*glc, luteolin *O-*hex (2), *O-*methylluteolin *O-*glcr, sulfuretin, **luteolin**, **genistein**, luteolin methyl ether	dyer's broom + traces of young fustic	
Church of St Andrew (Monastery Church of the Poor Clares)	No. 245	chasuble, main textile	Europe, the 17th cent. 3	green	**genistin**, **luteolin 7-*****O-*****glc**, genistein *O-*hex, luteolin *O-*hex (1), **apigenin 7-*****O-*****glc**, luteolin *O-*hex (2), *O-*methylluteolin *O-*glcr, **luteolin, genistein**, luteolin methyl ether, isatin, **indigotin**	dyer's broom + indigo/woad	
Church of St Mark	No. 249	chasuble, textile from sides	Europe, the 17th cent. 3	green	**luteolin di-*****O-*****hex**, **luteolin 7-*****O-*****glc**, **apigenin 7-*****O-*****glc**, luteolin *O-*hex (2), *O-*methylluteolin *O-*glcr, **luteolin**, apigenin, diosmetin, isatin, **indigotin**, indirubin	weld + indigo/woad	
Basilica of Holy Trinity (Monastery Church of the Dominicans)	No. 250	stole, main textile	Europe, the 17th cent. 3	yellow	**luteolin di-*****O-*****hex**, **luteolin 7-*****O-*****glc**, apigenin 7-*O-*glc, luteolin *O-*hex (2), **luteolin**, apigenin, diosmetin	weld	
Basilica of Holy Trinity (Monastery Church of the Dominicans)	No. 260	stole, main textile	Europe, the 17th cent. 3	yellow	braz3, fustin, braz5, luteolin 7-*O-*glc, fisatin, **sulfuretin**, madder1	young fustic	traces of synthetic dye
Church of St Peter and St Paul	No. 269	stole, silk main textile	Europe, the 17th cent. 3	green	luteolin *C-*hex, carminic acid, **genistin**, **luteolin 7-*****O-*****glc**, genistein *O-*hex, luteolin *O-*hex (1), **apigenin 7-*****O-*****glc**, luteolin *O-*hex (2), luteolin, **genistein**, apigenin, diosmetin, isatin, **indigotin**, indirubin	dyer's broom + indigo/woad	
Church of St Anne	No. 271	maniple, main textile	Europe, the 17th cent. 3	orange	braz1, braz2, braz3, **braz4**, **braz5, braz6**, **bixin**	brazilwood + annatto	
Church of the Visitation of the Blessed Virgin Mary (Monastery Church of the Carmelites)	No. 287	chasuble, textile from orphrey	N/A, c. 1700 3	green	**genistin**, **luteolin 7-*****O-*****glc**, **apigenin 7-*****O-*****glc**, luteolin *O-*hex (2), *O-*methylluteolin *O-*glcr, **luteolin, genistein**, apigenin, isatin, **indigotin**	dyer's broom + indigo/woad	
Church of the Assumption of the Holy Mother and St Venceslaus (Monastery Church of the Cistercians)	No. 288	chasuble, orphrey and around-neck-opening textile	Europe, the 17th cent. 3	green	**genistin**, **luteolin 7-*****O-*****glc**, luteolin *O-*hex (1), apigenin 7-*O-*glc, luteolin *O-*hex (2), *O-*methylluteolin *O-*glcr, **luteolin**, genistein, isatin, **indigotin**	dyer's broom + indigo/woad	
Church of St Francis of Assisi (Monastery Church of the Franciscans)	No. 292	chasuble, main textile	Europe, the 17th cent. 3	green	**genistin**, **luteolin 7-*****O-*****glc**, genistein *O-*hex, luteolin *O-*hex (1), **apigenin 7-*****O-*****glc**, luteolin *O-*hex (2), *O-*methylluteolin *O-*glcr, **luteolin, genistein**, luteolin methyl ether, apigenin, isatin, **indigotin**, indirubin	dyer's broom + indigo/woad	
Church of the Conversion of St Paul (Monastery Church of the Lazarists)	No. 293	stole, main textile	Europe, the 17th cent. 3	green	**luteolin 7-*****O-*****glc**, **luteolin*****O-*****glcr(1)**, luteolin *O-*glcr (2), *O-*methylluteolin *O-*glcr, **luteolin**, apigenin, isatin, **indigotin**	sawwort + indigo/woad	
Church of St Michael the Archangel and St Stanislaus Bishop and Martyr (Monastery Church of the Pauline Fathers)	No. 298	chasuble, textile from sides	Near East, the 15th cent. 4	green	chlorogenic acid, carminic acid, **luteolin 7-*****O-*****glc, luteolin*****O-*****glcr (1)**, **luteolin*****O-*****glcr(2)**, *O-*methylluteolin *O-*glcr, **luteolin**, **apigenin**, acacetin, isatin, orchil1, orchil2, **indigotin**, indirubin	sawwort + indigo/woad + traces of orchil	
Church of St Francis of Assisi (Monastery Church of the Franciscans)	No. 299	stole, main textile	Near East, the 16th cent. 4	green	**rutin**, **hyperoside**, **luteolin 7-*****O-*****glc**, **kaempferol*****O-*****hex-dhex**, kaempferol 3-*O-*glc, unknown (577), **luteolin, datiscetin**, unknown (621), unknown (591), biochanin A, unknown (299), madder2, madder3, isatin, **indigotin**, indirubin	unknown yellow flavonoid dye II + bastard hemp (unknown yellow flavonoid dye III) + indigo/woad + traces of madder (of unknown origin)	
Church of St Francis of Assisi (Monastery Church of the Franciscans)	No. 299	chasuble, textile from sides	Near East, the 16th cent. 4	green	**rutin**, **hyperoside, luteolin 7-*****O-*****glc, kaempferol*****O-*****hex-dhex**, kaempferol 3-*O-*glc, unknown (577), **luteolin, datiscetin**, unknown (621), unknown (591), biochanin A,**unknown (299)**, madder2, madder3, isatin, **indigotin**, indirubin	unknown yellow flavonoid dye II + bastard hemp (unknown yellow flavonoid dye III) + indigo/woad + traces of madder (of unknown origin)	
Church of St Francis of Assisi (Monastery Church of the Franciscans)	No. 317	chasuble, orphrey and around-neck-opening textile	Near East, the 17th cent. 4	green	**luteolin di-*****O-*****hex**, **luteolin 7-*****O-*****glc**, **luteolin*****O-*****hex (1), apigenin 7-*****O-*****glc, luteolin*****O-*****hex (2)**, *O-*methylluteolin *O-*glcr, **luteolin**, apigenin, diosmetin, acacetin, isatin, **indigotin**, indirubin	weld + indigo/woad	
Church of the Assumption of the Blessed Virgin Mary (Monastery Church of the Camaldolese Monks)	No. 320	stole, main textile	Near East, the 17th cent. 4	green	luteolin *C-*hex, **rutin**, **hyperoside**,**luteolin 7-*****O-*****glc**, **kaempferol*****O-*****hex-dhex**, kaempferol 3-*O-*glc, **ellagic acid**, **unknown(577)**, **luteolin**, **datiscetin**, **unknown(621)**, **unknown (591)**, biochanin A, **unknown (299)**, isatin, **indigotin**, indirubin	unknown yellow flavonoid dye II + bastard hemp (unknown yellow flavonoid dye III) + indigo/woad	
Church of the Annunciation to the Blessed Virgin Mary (Monastery Church of the Capuchins)	No. 321	chasuble, textile from front sides	Near East, the 17th cent. 4	green	**luteolin di-*****O-*****hex**, **luteolin 7-*****O-*****glc**, luteolin *O-*hex (1), **apigenin 7-*****O-*****glc**, luteolin *O-*hex (2), *O-*methylluteolin *O-*glcr, **luteolin**, apigenin, diosmetin, isatin, **indigotin**	weld + indigo/woad	
Church of St Anne	No. 322	burse, main textile	Near East, the 17th cent. 4	green	**luteolin di-*****O-*****hex**, **luteolin 7-*****O-*****glc**, luteolin *O-*hex (1), apigenin 7-*O-*glc, luteolin *O-*hex (2), **luteolin**, apigenin, diosmetin, isatin, **indigotin**, indirubin	weld + indigo/woad	
Basilica of Holy Trinity (Monastery Church of the Dominicans)	No. 326	chalice velum, main textile	Near East, the 17th cent. 4	green	luteolin/kaempferol *O-*hex-dhex-dhex, quercitrin, rhamnocitrin *O-*(hex-dhex-dhex), **luteolin**, kaempferol, biochanin A, **rhamnocitrin**, **emodin**, isatin, **indigotin**, indirubin	Persian berries + indigo/woad	
Church of the Annunciation to the Blessed Virgin Mary (Monastery Church of the Capuchins)	No. 327	chalice velum, main textile	Near East, the 17th cent. 4	greenish blue	rutin, luteolin/kaempferol *O-*hex-dhex-dhex, kaempferol *O-*hex-dhex, apigenin 7-*O-*glc, rhamnocitrin *O-*(hex-dhex-dhex), luteolin, biochanin A, biochanin A, rhamnocitrin, **emodin**, isatin, **indigotin**, indirubin	Persian berries + indigo/woad	
Church of the Visitation of the Blessed Virgin Mary (Monastery Church of the Carmelites)	No. 328	chasuble, main textile	Near East, the 17th cent. 4	green	**rutin**, hyperoside, **luteolin/kaempferol*****O-*****hex-dhex-dhex**, **kaempferol*****O-*****hex-dhex**, quercitrin, apigenin 7-*O-*glc, **rhamnocitrin*****O-*****(hex-dhex-dhex)**, **luteolin**, biochanin A, **rhamnocitrin**, **emodin**, isatin, **indigotin**	Persian berries + indigo/woad	traces of synthetic dyes
Church of St Barbara	No. 329	chasuble, main textile	Near East, the 17th cent. 4	green	chlorogenic acid, rutin, **hyperoside**, luteolin 7-*O-*glc, **kaempferol 3-*****O-*****glc**, **isorhamnetin 3-*****O-*****glc**, isatin, **indigotin**, indirubin	larkspur (unknown yellow flavonoid dye I) + indigo/woad	
Basilica of Holy Trinity (Monastery Church of the Dominicans)	No. 330	parura, main textile	Near East, the 17th cent. 4	green	chlorogenic acid, rutin, **hyperoside**, luteolin 7-*O-*glc, **kaempferol 3-*****O-*****glc**, **isorhamnetin 3-*****O-*****glc**, isatin, **indigotin**	larkspur (unknown yellow flavonoid dye I) + indigo/woad	
Basilica of Holy Trinity (Monastery Church of the Dominicans)	No. 332	chalice velum, main textile	Near East, the 17th cent. 4	green	chlorogenic acid, rutin, **hyperoside**, luteolin 7-*O-*glc, **kaempferol 3-*****O-*****glc**, **isorhamnetin 3-*****O-*****glc**, isatin, **indigotin**	larkspur (unknown yellow flavonoid dye I) + indigo/woad	
Basilica of Holy Trinity (Monastery Church of the Dominicans)	N/A (D-31#)	maniple, main textile	Europe, the 17th cent.	green	**luteolin*****C-*****hex**, **luteolin 7-*****O-*****glc**, **luteolin*****O-*****glcr(1)**, *O-*methylluteolin *O-*glcr, **luteolin**, isatin, **indigotin**	unknown yellow flavonoid dye II + indigo/woad	
Church of St Francis of Assisi (Monastery Church of the Franciscans)	N/A (F-11/3#)	chasuble, around-neck-opening textile	N/A	yellow	**braz1**, braz2, **braz3, braz4**, **braz5**, braz6, isatin, indigotin, **bixin**	brazilwood + annatto	
Church of St Francis of Assisi (Monastery Church of the Franciscans)	N/A (F-14/2#)	chasuble, textile from sides	Europe, the 17th cent.	green	**luteolin di-*****O-*****hex**, **luteolin 7-*****O-*****glc**, **apigenin 7-*****O-*****glc**, luteolin *O-*hex (2), **luteolin**, apigenin, diosmetin, isatin, **indigotin**, indirubin	weld + indigo/woad	
Church of St Francis of Assisi (Monastery Church of the Franciscans)	N/A (F-15/1#)	chasuble, textile from sides	Europe, the 17th cent.	green	**genistin**, **luteolin 7-*****O-*****glc**, genistein *O-*hex, luteolin *O-*hex (1), **apigenin 7-*****O-*****glc**, luteolin *O-*hex (2), *O-*methylluteolin *O-*glcr, **luteolin, genistein**, luteolin methyl ether, apigenin, isatin, **indigotin**, indirubin	dyer's broom + indigo/woad	
Church of St Francis of Assisi (Monastery Church of the Franciscans)	N/A (F-20/3#)	stole, main textile	Europe, the 16th-17th cent.	green	**luteolin di-*****O-*****hex**, **luteolin 7-*****O-*****glc**, luteolin *O-*hex (1), **apigenin 7-*****O-*****glc**, luteolin *O-*hex (2), *O-*methylluteolin *O-*glcr, **luteolin**, apigenin, diosmetin, isatin, **indigotin**, indirubin	weld + indigo/woad	
Corpus Christi Basilica	N/A (KBC-11/2#)	stole, lining	Europe, the 17th cent.	yellowish green	luteolin *C-*hex, **genistin**, **luteolin 7-*****O-*****glc**, luteolin *O-*hex (1), **apigenin 7-*****O-*****glc**, *O-*methylluteolin *O-*glcr, **luteolin**, **genistein**, apigenin, diosmetin, isatin, **indigotin**, **indirubin**	dyer's broom + indigo/woad	
Corpus Christi Basilica	N/A (KBC-18#)	stole, main textile	N/A	green	chlorogenic acid, luteolin *C-*hex, rutin, genistin, **luteolin 7-*****O-*****glc**, **luteolin *O*-glcr (1)**, **luteolin*****O-*****glcr(2)**, luteolin *O-*hex (1), luteolin *O-*hex (2), *O-*methylluteolin *O-*glcr, **luteolin**, **genistein**, luteolin methyl ether, apigenin, diosmetin, isatin, **indigotin**, indirubin	dyer's broom + sawwort + indigo/woad	
Church of the Visitation of the Blessed Virgin Mary (Monastery Church of the Carmelites)	N/A (KnP-8#)	chasuble, main textile	N/A	green	**luteolin di-*****O-*****hex**, **luteolin 7-*****O-*****glc**, **apigenin 7-*****O-*****glc**, luteolin *O-*hex (2), *O-*methylluteolin *O-*glcr, **luteolin**, apigenin, diosmetin, isatin, orchil1, orchil2, orchil3, orchil4, **indigotin**, indirubin	weld + indigo/woad + traces of orchil	
Church of the Conversion of St Paul (Monastery Church of the Lazarists)	N/A (M-7#)	chalice velum, main textile	Near East, the 17th cent.	violet	**carminic acid, luteolin di-*O*-hex**, braz5, **luteolin 7-*****O-*****glc**, **luteolin*****O-*****glcr (1)**, **luteolin*****O-*****glcr(2)**, **sulfuretin**, **luteolin**, apigenin	brazilwood + weld + young fustic + traces of cochineal (of unknown origin)	presence of synthetic dye, original fibre colour – yellow or orange

**bold and underlined** – main compounds; **bold**– secondary compounds; non-highlighted – minor compounds; glc – glucoside, hex – hexoside, glcr – glucuronide, braz1 − brazilwood compound 1, braz2 − brazilwood compound 2, braz3 − brazilwood compound 3, braz4 − brazilwood compound 4, braz5 − brazilwood compound 5, braz6 − brazilwood compound 6, madder1 – madder compound 1, madder2 – madder compound 2, madder3 – madder compound 3, orchil1 – orchil compound 1, orchil2 – orchil compound 2, orchil3 – orchil compound 3, orchil4 – orchil compound 4;

⁎ detailed data presented in “KATALOG TKANIN z zasobów kościelnych Krakowa z czasów od XV do końca XVII” (in Polish), Natalia Krupa (ed.), Kraków (in printing); textile origin and dating have been established by: ^1^ N. Krupa,

2 A. Warzecha

3 K. Moskal

4 B. Biedrońska-Słota

# initial inventory number of the object

## 2 Experimental Design, Materials, and Methods

The 89 silk fibres (yellow, orange, brown and green) were taken from 15th- to 17th-century silk textiles used in vestments belonging to the collections of seventeen Krakow churches (all samples are listed in Table 1).

Brown and green fibres were extracted twice, using two extraction methods consecutively, the first one with dimethylsulfoxide (DMSO), and the second one with acidic-methanol extractant. Yellow and orange fibres were extracted only with the second procedure. Extraction procedure have been described in detail by Lech [1].

Separation of the colorants was carried out using a 1220 Infinity II LC Systems (Agilent Technologies, USA), whereas their identification was achieved with two spectrophotometric detectors, a 1220 Compact Variable Wavelength Detector (Agilent Technologies, Germany) and a 1200 Variable Wavelength Detector (Agilent Technologies, Germany), as well as with a 6460 Triple Quad tandem mass spectrometric detector with electrospray ionization Jet Stream ion source (Agilent Technologies, USA). Analytes were separated by a Zorbax SB-Phenyl rapid resolution column (4.6 × 150 mm, 3.5 μm, 80 Å, Agilent Technologies) protected by a Zorbax SB-Phenyl precolumn (4.6 × 12.5 mm, 5.0 µm, Agilent Technologies). Spectrophotometric detection was performed at various wavelengths depending on the analyzed dye (280, 400, 450, 480, 500, 550, 580 or 600 nm). Mass spectrometric data were acquired in dynamic multiple reaction monitoring (dMRM) modes of negative and positive ions. Parameters of the method were described by Lech [1]. The analyses were controlled and processed by a MassHunter Workstation software (Agilent Technologies, USA).

## Declaration of Competing Interest

The author declares that she has no known competing financial interests or personal relationships which have, or could be perceived to have, influenced the work reported in this article.
